# Effects of Telerehabilitation Combining Diaphragmatic Breathing Re-Education and Shoulder Stabilization Exercises on Neck Pain, Posture, and Function in Young Adult Men with Upper Crossed Syndrome: A Randomized Controlled Trial

**DOI:** 10.3390/jcm13061612

**Published:** 2024-03-11

**Authors:** Gyeong-Hyeon Jeong, Byoung-Hee Lee

**Affiliations:** 1 Graduate School of Physical Therapy, Sahmyook University, Seoul 01795, Republic of Korea; jgh10108@naver.com; 2 Department of Physical Therapy, Sahmyook University, Seoul 01795, Republic of Korea

**Keywords:** shoulder, neck pain, posture, telerehabilitation, breathing exercises

## Abstract

**Background:** Forward head posture and rounded shoulder posture are common postural variants found in upper crossed syndrome, which can lead to limited neck mobility, respiratory problems, and other issues. The purpose of this study was to investigate the effects of telerehabilitation, combining diaphragmatic breathing re-education and shoulder stabilization exercises, on young men with upper crossed syndrome during the COVID-19 pandemic over 4 weeks. **Methods:** The study included 37 participants aged in their 20s and 30s who were randomly divided into two groups. The experimental group received diaphragmatic breathing re-education and shoulder stabilization exercises, while the control group only underwent shoulder stabilization exercises. Both groups were trained three times a week for four weeks using telerehabilitation. The comparison of within-group pre–post differences in the experimental and control groups was conducted using a paired *t*-test, while the effects of treatment were assessed using repeated-measures analysis of variance. **Results:** After 4 weeks, both groups showed significant improvements in the pain pressure threshold of the upper trapezius, craniovertebral angle, round shoulder posture, shoulder tilt degree, neck disability index, and closed kinetic chain upper extremity stability test (all *p* < 0.05). The results showed a significant difference between the Time effect (*p adj* < 0.05/4) for both sides of PPT, CVA, and STD and both sides of RSP, NDI, and CKCUEST, and an interaction between the Time × Group effects (*p adj* < 0.05/4) for the Rt. PPT, CVA, and STD. **Conclusions:** These findings suggest that the telerehabilitation training group, which included diaphragmatic breathing re-education and shoulder stabilization exercises, was more effective in improving Rt. PPT, CVA, and STD in males with UCS.

## 1. Introduction

Upper crossed syndrome (UCS), a condition first described by Janda, is characterized by a shortening of the superficial neck flexor muscles and a relaxation of the deep neck flexor and extensor muscles [1]. There are several ways to evaluate the alignment of the cervical vertebrae, with forward head posture (FHP) being a common symptom of UCS [2]. FHP caused by bending of the upper and lower cervical vertebrae can be measured using the craniovertebral angle (CVA) [3]. A CVA of 52° or less indicates the presence of FHP [4]. Symptoms of FHP include fatigue, limited neck mobility, dysfunction of jaw joints, chronic migraines, numbness in the hands, and muscle tremors [5,6,7].

It can also impair diaphragmatic function and movement, leading to inefficient contraction of the abdominal muscles, decreased lung capacity [8], and a negative impact on respiratory function [9]. Round shoulder posture (RSP) is another symptom of UCS, defined as an acromion process higher than 2.5 cm when lying flat on the floor [10]. RSP is caused by a shortening of the pectoralis minor muscle and a relaxation of the middle and lower trapezius (LT) muscles, resulting in symptoms such as numbness of the hands, back pain, restricted shoulder mobility, and breathing changes [11,12]. Increased cervical and thoracic curvature and sloping posture have been shown to affect scapular movement, shoulder muscle strength, and shoulder range of motion (ROM) [13,14,15]. To correct FHP and RSP, which are the main postural abnormalities observed in UCS, it is necessary to strengthen the serratus anterior (SA), middle, and LT muscles. A common exercise for this purpose is shoulder stabilization. Resistance bands can be used in combination with posture programs that include strengthening and stretching exercises to correct FHP. Resistance bands can improve functional activity, strength, and balance [16]. In individuals with UCS, certain muscles, such as the sternocleidomastoid (SCM), scalene, pectoralis minor, and upper trapezius (UT), can become overactive during breathing, leading to reduced activation of the diaphragm, which is the primary dynamic muscle involved in breathing [17].

The diaphragm is a thin, dome-shaped muscle–tendon structure, approximately 2–4 mm thick, and is a vital dynamic muscle for breathing. As Stone noted, ‘the diaphragm is one of the most remarkable areas of the body in that it has so much influence, and the consequences of its dysfunction can be anywhere from the head to the toe’ [18]. Diaphragmatic breathing (DB) re-education has the effect of strengthening muscle strength and improving the lung capacity of the deep neck flexor muscles, leading to positive results for posture control in FHP and RSP patients [19]. The purpose of using DB re-education training is to alleviate UCS by reducing excessive activity in the neck muscles among UCS patients, restoring regular diaphragmatic function and aiming for pain alleviation, prevention of secondary damage, and nerve stabilization, along with various other advantages.

Telerehabilitation introduces an innovative approach to delivering rehabilitation services from a distance using various communication technologies like telephone, videoconferencing, computer software, and mobile applications. Healthcare professionals increasingly integrate telerehabilitation into their multidisciplinary treatment strategies, particularly when traditional healthcare facilities such as hospitals and clinics are inaccessible, causing disruptions to supervised healthcare. Despite not fully replicating the one-on-one treatment experience offered in conventional settings, telerehabilitation proves its effectiveness, as evident through factors like cost-efficiency, resource optimization, and time management. This emerging field plays a pivotal role in expanding access to quality rehabilitation services while effectively overcoming geographical and logistical barriers [20].

The systematic review by Egmond M.A. et al. [21] reported significant effects on the quality of life and satisfaction of patients who underwent cardiac surgery, orthopedic surgery, and oncological surgery in the abdominal, thoracic, and cervical regions. The experimental group, which received physical therapy with telerehabilitation for postoperative management, demonstrated a notable improvement compared to the control group that did not receive physical therapy with telerehabilitation. Additionally, Jachak et al. [22] reported that physical therapy with telerehabilitation activated due to COVID-19 can reduce psychological risks for patients, enhance motivation for treatment, elevate the level of care for individuals with physical and mental health issues, and lower hospital costs. This review article includes studies that experimented with telerehabilitation for patients with acute and chronic musculoskeletal disorders, cardiovascular diseases, and neurological conditions.

However, there has been no prior research investigating the effects of telerehabilitation, including DB re-education and SSE, in young adult men with UCS. This study aims to investigate whether a group of young adult men with UCS, trained through DB re-education and SSE through telerehabilitation, demonstrates greater improvement in neck pain, posture, and function compared to a group that only receives SSE through telerehabilitation during the COVID-19 pandemic.

## 2. Materials and Methods

### 2.1. Subjects

This study recruited participants for one month from 31 January 2023 to 28 February 2023. In total, 40 generally healthy young men in their 20s and 30s who lived in Seoul were selected. The inclusion criteria for this study were age in the 20s and 30s, no history of outpatient or hospital treatment, no musculoskeletal or neurological diseases, no back pain during DB re-education and training, no shoulder pain during SSE, a CVA of less than 52°, and an acromion process higher than 2.5 cm when lying flat on the floor. The exclusion criteria were inability to continue plank exercise due to back or shoulder pain, diagnosis of neck or lumbar disc herniation, and a history of neck or lumbar surgery within the past 6 months. All participants provided written informed consent after receiving a full explanation of the study purpose, expected outcomes, and potential muscle soreness after exercise.

This study was approved by the Sahmyook University Institutional Review Board (approval number: 2022-12-008-001) and the Clinical Research Information Service (KCT0008383) for the Human Studies Committee and was conducted according to the ethical principles of the Declaration of Helsinki. All participants provided informed consent for inclusion in the study after fully understanding the objectives and procedures to be performed.

### 2.2. Experimental Procedure

Participants were selected for this study based on the inclusion and exclusion criteria. Before recruiting participants for this study, we performed a power analysis using G*Power version 3.1.9.7 (Franz Faul, University Kiel, Kiel, Germany, 2020). According to a previous study [5,23,24,25,26,27], the effect size for PPT, CVA, RSP, STD, NDI, and CKCUEST was 0.506, 0.317, 0.899, 0.707, 0.078, and 0.412, respectively. This study calculated the effect size by averaging the effect sizes from previous studies. The sample size for this study was determined with an effect size of 0.486, a standard deviation of 0.266, a significance level of 0.0125 (0.05/4), a power of 0.95, and a number of groups of 2, resulting in a required sample size of 12 per group. Because the estimated target sample size was 24, we recruited 40 participants for this experiment [20].

Pretests were performed one week before the program started. A total of 40 participants who initially indicated their willingness to participate were randomly assigned using SPSS to either the experimental group that performed DB re-education and shoulder stabilization exercises (DB + SSE group; *n* = 20) or the control group that performed only SSE (*n* = 20).

Before the experiment, participants were asked to report their general characteristics, such as age, height, and weight, directly through a questionnaire. The pain pressure threshold (PPT) of the bilateral UT and CVA, the shoulder tilt degree (STD), the bilateral RSP, the Korean version of the neck disability index (NDI), and the closed kinetic chain upper extremity stability test (CKCUEST) were measured. The participants did not perform warm-up or cool-down exercises before and after receiving treatment three times a week for 4 weeks during telerehabilitation.

In the first week, the researchers will provide direct supervision for education and training, and from the second week onward, DB re-education and SSE will be conducted remotely via videoconferencing software, specifically Zoom^®^ (version 5.13.0, Zoom Video Communications, San Jose, CA, USA), a real-time videoconferencing software, for 4 weeks. The control group will exclusively perform SSE, following the same supervision format as the intervention group, with direct researcher oversight during the first week and remote online Zoom sessions for SSE starting from the second week onwards.

**Figure 1:**
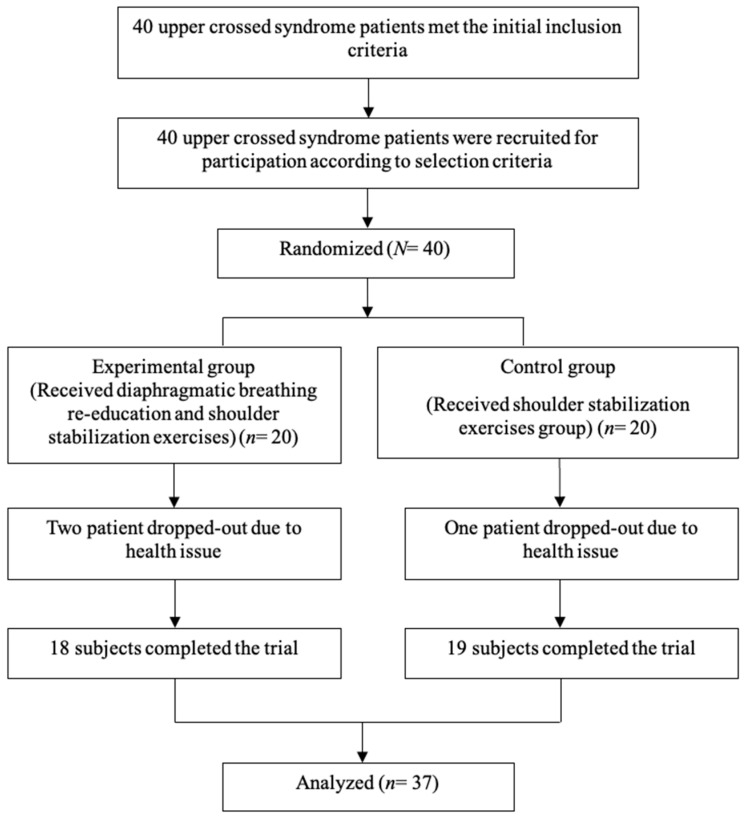
Flow diagram of the total experimental procedure.

The same physical therapist conducted all measurements to ensure consistency. After four weeks of the intervention, the PPT of the bilateral UT and the CVA, STD, bilateral RSP, NDI, and CKCUEST scores were measured. The primary outcomes of this study are the PPT of both upper trapezius muscles, CVA, and the Korean version of the neck disability index. The secondary outcomes are acromion’s tilt degrees and closed kinetic chain upper extremity stability test. The study protocol is illustrated in the following chart (Figure 1).

### 2.3. Training Program

#### 2.3.1. Diaphragmatic Breathing Re-Education Program

**Figure 2:**
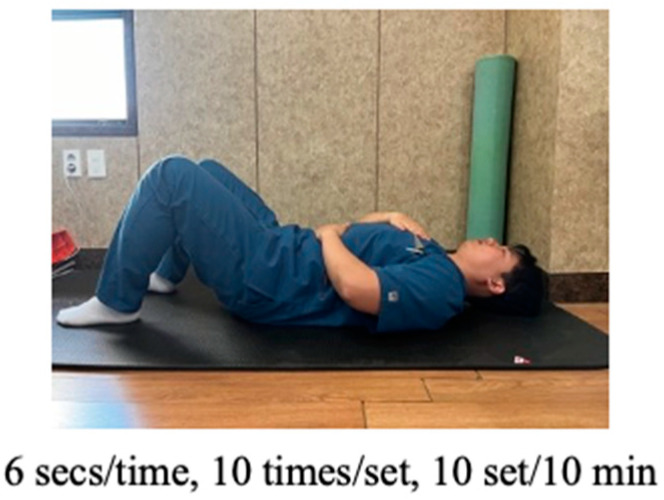
Diaphragmatic breathing re-education.

Diaphragmatic breathing (DB) is typically performed in the supine position by breathing in slowly and deeply through the nose using the diaphragm, with minimal movement of the chest. One hand is placed on the chest and the other on the belly to ensure proper technique. After deep inhalation through the nose, individuals are instructed to exhale through the mouth while focusing on the contraction of the abdominal muscles. During practice, the experimental group was instructed to focus on contracting the diaphragm while keeping the chest as still as possible and inhaling and exhaling for approximately six seconds each. According to the protocol, the intervention consisted of conducting a total of 10 sets of DB exercises, each lasting 1 min (Figure 2).

#### 2.3.2. Shoulder Stabilization Exercises Program

The intervention involved performing plank exercises and strengthening exercises for the rotator cuff, SA, and LT muscles. For the rotator cuff muscle exercises, the participants were in a seated position. They instructed to place a loop band (GOMUNARA Loop Band, Phase 1. 60 × 5 cm^2^ size, 0.35 mm thickness, Republic of Korea) on both hands, positioning it shoulder-width apart. The starting position required the shoulders to be straight and the elbows to be bent to 90°, with the forearms in a supinated position. To perform this exercise, only the rotator cuff muscles were used to work against the resistance of the loop band and simultaneously perform shoulder external rotation exercises. Each set consisted of 10 repetitions, and a total of five sets were completed. The break time between sets was set to 30 s.

For the SA muscle exercises, the participants were in a seated position and started by bending their elbows to 90° as if they were beginning to train the rotator cuff muscles. The participants then placed both wrists in the provided loop band to keep the forearms in a neutral position and keep the band tight at shoulder width. The shoulder joint was then flexed to a 90° angle, and the elbow joint was extended by raising both arms above the head while maintaining shoulder width. Each set consisted of 10 repetitions, and a total of five sets were completed. The break time between sets was set to 30 s.

For the LT muscle exercises, the participants were in a seated position and instructed to raise their arms in a Y-shape while sitting directly on a chair with their thumbs facing backward. They were instructed to engage in isometric contractions of the LT muscle, focusing on bringing the scapula and humerus together. The contraction was held for 10 s, followed by a 10-s break. A total of five sets were completed.

**Figure 3:**
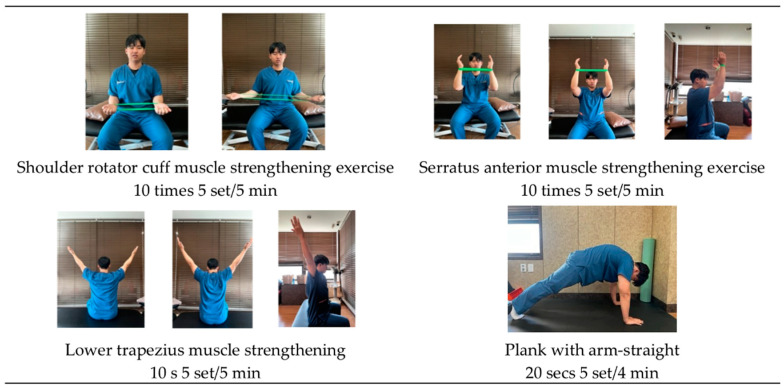
Shoulder stabilization exercise program.

For shoulder and core muscle exercises, the participants were instructed to be prone on the floor and position their arms shoulder-width apart. The participants were then asked to extend their arms and hold a position similar to that of a push-up. Each set lasted 20 s, and a total of five sets were completed. The break time between sets was set to 30 s (Figure 3).

#### 2.3.3. Outcome Measures

To measure the PPT, participants were instructed to sit on a chair with hip and knee joints flexed at 90° degrees. A measuring instrument was applied to the central area of the upper trapezius muscle, and pressure was applied at a rate of 1 kg/s until the participant felt pain. Upon experiencing pain, the participant vocalized ‘ah’, and the examiner recorded the corresponding numerical value. The measurements are conducted separately for the right and left sides [28]. A Wagner’s FDX-25 (Digital Algometer, Greenwich, CT, USA) was used to evaluate the PPT of the UT muscle in a sitting position by recording the average of three measurements at the center of the UT muscle [29]. In a previous study, the FDX-25 has a high reliability of 0.82–0.97 and has established validity [30,31].

In this study, the smartphone app ‘FHPapp’ (Pyeongtaek, Republic of Korea), which provides users with the ability to measure CVA using a side-view photograph, was used to evaluate the CVA to assess FHP. Participants were examined in a straight standing posture, with a horizontal line established based on the C7 spinous process. The angle between the C7 spinous process and the ear tragus was measured. The distance between the participants and the smartphone was maintained at 1.5 m to ensure standardization. The CVA measurement was conducted only on the right side of the participants.

CVA measurements were performed twice, and the average was recorded. The intra-rater reliability of the FHPapp was found to be 0.88 [32], while previous research found the reliability of the CVA to be 0.88 [33].

In a supine position, the height of the RSP was measured using a digital caliper (0–150 mm Stainless Hardened), from the base to the anterior tip of the participant’s acromion process. Measurements were taken three times on both the right and left sides, and the averages were recorded. The reliability of this method is 0.89–0.99 [34].

They were measured in a standing position using the AcuAngle^®^ inclinometer (Baseline AcuAngle Inclinometer, New York, NY, USA) to measure shoulder tilt degree (Shoulder Height Discrepancy Test) in a standing position. After placing both ends of the inclinometer on the participant’s acromion process, the tilt angle was measured in one direction. The STD for all participants leaned towards the right, and the average was recorded after three replicate measurements. The reliability of the measurement tool used in this study is 0.98 [35].

Neck dysfunction was evaluated using the Korean version of the NDI, which consists of 10 questions on functional activity, symptoms, and concentration. The NDI score was calculated by summing the responses to each item, with a higher score indicating greater neck-related dysfunction. Participants received the Korean version of the neck disability index (NDI) printed on paper, and they completed it both before and after the experiment. The intraclass-correlation coefficient (ICC) of test–retest reliability of the Korean version of NDI is 0.93 [36].

To assess the level of upper extremity stability, the CKCUEST proposed by Davies (2000) was used. This experiment involves marking a 90 cm-wide area on the floor and assuming a push-up position after spreading both hands at the same width. The testing procedure measures one repetition as lifting one hand, touching the marked point on the opposite side, and returning to the original position, counting as one cycle. For example, if the right hand first touches the marked point on the left and returns, it is recorded as one repetition; then, if the left hand subsequently touches the marked point on the right and returns, it is recorded as two repetitions. In this study, the experiment was conducted with a width set to 80 cm (width of the shoulder joint) [37]. The participants took turns placing one hand on top of the other, and the action of placing one hand on top of the other was counted once. The number of times the participants performed this action for a maximum of 15 s was measured by repeating it for a total of 15 s [38]. To ensure proper posture maintenance, the participants received detailed instructions before the test. A one-minute break was taken between tests, and the average of the two measurements was utilized. In a previous study involving 40 participants, this test demonstrated high reliability, with an ICC of 0.97 [39].

#### 2.3.4. Data Analysis

All statistical analyses were performed using the SPSS version 25.0 (SPSS Inc., Chicago, IL, USA), which was used to calculate the means and standard deviations. All variables were normally distributed. Descriptive statistics were used to describe the general characteristics of the participants. To compare differences before and after intervention, a paired t-test was used. An independent samples t-test was utilized to compare differences between groups. The effects of treatment were analyzed by repeated-measures analysis of variance. To investigate inter-group differences, a total of 4 independent tests were conducted, with a statistical significance level set at α = 0.05. To address errors arising from multiple comparisons, the Bonferroni correction was applied to determine the significance level. Therefore, the significance level for inter-group effect testing in this study was set at 0.0125 (0.05/4) for comparative analysis.

## 3. Results

The subjects of this experiment consisted of a total of 37 individuals. A homogeneity test of the general characteristics of the participants revealed no significant differences in the average age (*p* = 0.824), weight (*p* = 0.818), or height (*p* = 0.821) between the two groups (Table 1).

The experimental group that received DB re-education and the SSE program through telerehabilitation showed significant improvement in PPT for both the left (*p* < 0.001) and right trapezius (*p* < 0.001). The control group, who only performed SSE through telerehabilitation, also showed significant improvement in PPT for both the left (*p* < 0.001) and right sides (*p* = 0.047). The results showed a significant difference between the Time × Group interaction effect (*p adj* < 0.05/4) for Rt. sides of PPT (*p* = 0.002) (Table 2).

Both the experimental and control groups showed significant improvements in CVA (*p* < 0.001, *p* < 0.001, respectively), STD (*p* < 0.001, *p* = 0.030, respectively), Lt. RSP (*p* < 0.001, *p* = 0.001, respectively), and Rt. RSP (*p* < 0.001, *p* = 0.001, respectively). The results showed a significant difference between the Time effect (*p adj* < 0.05/4) for CVA (*p* < 0.001), STD (*p* < 0.001), and both sides of RSP (*p* < 0.001, *p* < 0.001, respectively) and a significant Time × Group effect (*p adj* < 0.05/4) for the CVA, STD (*p* = 0.005, *p* = 0.004, respectively) (Table 3).

Both the experimental group and control group showed significant improvements in the NDI and CKCUEST scores (*p* < 0.001, *p* < 0.001, respectively). The results showed a significant difference between the Time effect (*p adj* < 0.05/4) for NDI and CKCUEST (*p* < 0.001, *p* < 0.001, respectively) and no interaction between the Time × Group effect (*p adj* > 0.05/4) for the NDI and CKCUEST (*p* = 0.529, *p* = 0.382, respectively) (Table 4).

## 4. Discussion

This study aimed to investigate whether a group of young adult men with upper crossed syndrome, who participated in DB re-education and SSE through telerehabilitation, demonstrated effects on neck pain, function, and posture compared to a group that only received SSE through telerehabilitation. Thirty-seven participants were divided into two groups, with three dropouts. Results indicated that the experimental and control groups showed significant improvements in all outcome measures (*p* < 0.05) from baseline to postintervention. The results showed a significant interaction between the Time × Group effect (*p adj* < 0.05/4) for the Rt. PPT, CVA, and STD.

These findings suggest that among males with UCS, the telerehabilitation training group combining diaphragmatic breathing re-education and shoulder stabilization exercises was more effective in improving Rt. PPT, CVA, and STD compared to the group that only underwent telerehabilitation focusing on shoulder stabilization exercises.

To reduce pain through breathing therapy, it may engage additional central pain control mechanisms. Previous research has shown that slow and calm diaphragmatic breathing can activate the vagus nerve, a critical pathway for transmitting and modulating sensory information between the brain and peripheral tissues [40]. Lee et al. (2015) assigned participants with neck pain to two groups, the shoulder stabilization exercise group (SSEG) and the control group [41]. The study found that the SSEG experienced a significant reduction in PPT in the UT muscles after exercise (*p* < 0.05) and had significantly lower NDI scores than the control group (*p* < 0.05). Dareh-deh et al. (2022) [42] demonstrated that a combination of DB re-education and other physiotherapy interventions resulted in a significant improvement in the strength of the deep neck flexors and forced vital capacity (FVC) in the experimental group. The results indicated that the intervention group, which included a combination of routine therapeutic techniques and respiratory exercises, showed significant differences in diaphragm muscle activation (*p* = 0.03), respiratory balance (*p* = 0.04), and the number of breaths (*p* = 0.02) when compared to the control group which received routine therapeutic exercise alone. Performing the suggested breathing exercises effectively resulted in changes in SCM activity when comparing the two groups. This study indicates that mediating respiratory feedback exercises are more efficient in enhancing SCM activity, neck flexor function, and NDI compared to those in the control group. This suggests that the inclusion of respiratory exercises in the management of neck pain may be beneficial for improving posture, muscle activity, and respiratory balance.

In a prior study investigating the effects of neck positioning on respiratory capacity, researchers observed a significant decrease in Sniff nasal inspiratory pressure (SNIP) scores when subjects sat with FHP compared to upright sitting (*p* < 0.001). This study highlighted an immediate impact of head–neck position changes on respiratory function, notably marked by reduced diaphragm strength. It underscores the substantial role that head positioning plays in the mechanical functioning of the respiratory system, even in healthy individuals, potentially due to temporary phrenic nerve compression, leading to reduced neural activity and subsequent diaphragmatic weakening [9]. Kim et al. (2017) found a significant positive correlation between CVA and vital capacity (VC), FVC, and forced expiratory volume in 1 s (FEV1), and a significant negative correlation between CVA and SCM activity ratios (r = 0.580, *p* = 0.000; r = 0.525, *p* = 0.002; r = 0.540, *p* = 0.001; and r = −0.361, *p* = 0.039, respectively) [43]. These results indicate the possibility that FHP causes low respiratory function and suggest that correcting head posture can prevent respiratory dysfunction. Therefore, if excessive smartphone usage is observed, it is crucial to incorporate the DB training and SSE interventions implemented in this study into interventions for individuals with FHP, while also addressing the potential negative effects of prolonged smartphone use. Kang et al.’s (2016) study confirmed a significant difference in SCM activity and NDI outcomes between the control group and the experimental group with feedback respiratory exercises. These findings align with the positive impact of the DB re-education applied in this study on NDI improvement [44].

The diaphragm plays a vital role in numerous physiological processes, including vocalization, swallowing, respiration, postural stability, defecation, micturition, and parturition, primarily by regulating intra-abdominal pressure. Impairment of the diaphragm has been associated with a range of disorders, including respiratory insufficiency, exercise intolerance, sleep disturbances, and potentially life-threatening conditions [45,46]. However, the phrenic nerve responsible for sensory and motor control of the diaphragm originates from C3–C5 and occasionally C6 nerve roots, coursing through the anterior scalene fascia. Notably, the overuse of the anterior scalene muscle, commonly observed in individuals with FHP, can lead to gradual compression of the phrenic nerve over time, potentially resulting in trophic changes and diaphragmatic dysfunction [47]. Therefore, through the DB re-education implemented in this study, proper breathing can be achieved, leading to improvements in forward head posture and neck pain. Additionally, it can help prevent secondary dysfunction due to diaphragmatic impairment and alleviate phrenic nerve compression.

Shaw et al. found that combining DB with aerobic exercise (AE) can effectively improve pulmonary function, cardiorespiratory fitness, and respiratory muscle strength in patients with moderate-persistent asthma [48]. The AEDB group was as effective as the AE group in increasing VO2max and FEV1, and more effective in increasing FVC. In addition to respiratory conditions, prior research, like the study conducted by Sahreen A. et al., has investigated the use of DB re-education in patients with musculoskeletal disorders such as chronic neck pain. These studies have shown significant improvements in neck flexor muscle strength and FVC (*p* < 0.05) [18]. Furthermore, Hamasaki’s review examined the effects of DB on various health outcomes, such as anxiety, quality of life in cancer patients, physical activity in heart failure patients, and migraine [49]. These findings collectively suggest that DB may serve as a valuable non-pharmacological intervention for enhancing respiratory function, quality of life, and overall well-being in patients with other diseases. These studies will enhance our research by addressing the gaps in our study, as we did not assess VO2 max, FVC, FEV1, or quality of life during DB re-education training.

Yaghoubitajani (2022) demonstrated that telerehabilitation of patients with neck–shoulder pain (NSP) due to COVID-19 led to significant improvements in NSP and postural angle, the activation of the UT and SA muscles, FHP, and RSP compared to the control group (*p* < 0.05) [50]. The findings of this study align with our results, demonstrating significant improvements in SA muscle activation, FHP, and RSP through SSE interventions in telerehabilitation. In addition, in a study by Shah (2022) [51], 428 patients who underwent telerehabilitation for spine pain during the COVID-19 pandemic lockdown were compared with 428 patients who underwent in-clinic multimodal rehabilitation treatment during the 6 months before the lockdown. Post-treatment, the telerehabilitation group showed a significant reduction in mean numeric pain rating scale (NPRS; MD = 1, *p* < 0.0001) and Oswestry disability index (ODI)/NDI (MD = 5.8, *p* < 0.0001) scores compared to the control group. Furthermore, there was a significantly higher percentage of patients in the telerehabilitation group who achieved a minimal clinically important difference (MCID) of ≥2 for NPRS (MD = 6%, *p* = 0.0007) and an MCID of ≥10 for ODI/NDI scores (MD = 7.5%, *p* = 0.005).

Therefore, the study concluded that telerehabilitation can significantly reduce pain and disability in patients with spine pain, and the results were better than those of in-clinic rehabilitation during the COVID-19 pandemic. This suggests the need to explore and test the efficacy and wider application of telerehabilitation for treating spine pain in the future [51]. These results have important implications for rehabilitation, indicating the potential benefits of utilizing telerehabilitation not only during pandemics but also in non-pandemic periods for treating patients with musculoskeletal disorders, including those with UCS.

The COVID-19 pandemic has reduced the availability of in-person patient treatments. Consequently, the future of rehabilitation therapy is expected to increasingly incorporate the telerehabilitation methods employed in this study. As supported by a wealth of research, telerehabilitation is not only convenient for patients with respiratory or musculoskeletal conditions but also for those dealing with neurological disorders and mobility limitations. Furthermore, with the potential for wider adoption of telerehabilitation, there are anticipated economic advantages and technological advancements. We hope that telerehabilitation will extend its reach beyond individual regions, offering treatment opportunities for patients in various cities and even countries. This has the potential to significantly expand the horizons of rehabilitation and enhance accessibility for patients worldwide. Finally, despite the relatively short duration, the 4-week experiment holds the potential to serve as a therapeutic period with anticipated long-term effects, as suggested by previous studies. Mustian KM et al. (2009) [52] implemented a home-based exercise program, incorporating both aerobic and resistance exercises for cancer patients undergoing radiation therapy. Following the 4-week experiment, a follow-up test was conducted 3 months later, revealing significant improvements in aerobic capacity, as evidenced by increases in daily steps walked (DSW) and daily minutes of resistance exercise (MRE). Moreover, there were notable enhancements in the number of resistance exercise days (RED) and in quality of life (QOL) compared to the control group (*p* < 0.05).

Furthermore, in a study conducted by Kanyilmaz T. (2022) [53], exploring virtual reality-based vestibular rehabilitation, significant improvements in vertigo, balance, and related factors were observed 6 months after a 4-week vestibular rehabilitation exercise compared to the control group (*p* < 0.05). Based on these studies, it can be inferred that the 4-week diaphragmatic breathing training and shoulder stabilization exercises conducted in this study may also yield long-term effects. Additionally, engaging in continuous training through a telerehabilitation program with temporal and spatial benefits could potentially lead to even more enhanced outcomes compared to the findings of this study.

This study had several limitations. First, only the PPT was used to measure the limitations of neck pain, and no other detailed measurements were recorded. The PPT and RSP were applied to both the left and right sides; however, the varying muscle tension and function of individual patients made it unclear whether the PPT and RSP had a greater effect on the more tensed side. Second, the CVA measurement tool (FHPapp) used in the study relied on participant cooperation, which could introduce biases in measurement and participant selection if body markers or measuring devices were not properly positioned or if data were misinterpreted. This bias can also affect measurements taken using a camera or phone application. Another limitation is that the study included only adult men; therefore, the results cannot be generalized to children, women, or older adults. Finally, there was insufficient time for the experimental period, which is an additional limitation of this study. Nevertheless, the results of this study suggest that DB re-education and SSE with telerehabilitation may effectively improve FHP and RSP in young men with upper crossed syndrome. Future research should investigate the potential of telerehabilitation services for rehabilitating a diverse range of patients with musculoskeletal disorders, as this has the potential to significantly improve patient outcomes, increase access to rehabilitation services, and enhance healthcare delivery.

## 5. Conclusions

The results of this study suggest that both the telerehabilitation training group with diaphragmatic breathing re-education and shoulder stabilization exercises and the shoulder stabilization exercise-based telerehabilitation training group showed improvements in neck pain, posture, and function in the postassessment compared to the preassessment. Additionally, for UCS patients, it was noted that implementing diaphragmatic breathing re-education was effective in improving Rt. PPT, CVA, and STD. However, the inclusion of diaphragmatic breathing re-education in the telerehabilitation intervention enhanced the program’s effectiveness in improving forward head posture and shoulder height balance. Therefore, the incorporation of diaphragmatic breathing and shoulder stabilization exercises with telerehabilitation is recommended for correcting upper crossed syndrome.

## Figures and Tables

**Table 1:** General characteristics of participants (*n* = 37).

Characteristics	Experimental Group (*n* = 18)	Control Group (*n* = 19)	*t* (*p*)
Age (years)	29.78 ± 1.89	29.95 ± 2.63	−0.224 (0.824)
Height (cm)	176.38 ± 4.59	176.84 ± 6.98	−0.232 (0.818)
Weight (kg)	81.22 ± 9.53	81.89 ± 8.41	−0.228 (0.821)

**Table 2:** Comparison of pain assessment outcomes (*n* = 37).

Variable		Experimental Group (*n* = 18)	Control Group (*n* = 19)	*t* (*p*)	Time F (*p*)	Group F (*p*)	Time × Group F (*p*)
Lt. PPT (kg/cm^2^)	Pretest	2.27 ± 0.39	2.20 ± 0.40	0.586 (0.561)			
	Post-test	2.75 ± 0.51	2.47 ± 0.30				
	Mean difference	0.47 ± 0.31	0.27 ± 0.18		81.214 (0.001)	1.839 (0.157)	5.377 (0.026)
	95% CI for difference	(0.31 to 0.62)	(0.19 to 0.36)				
	*t* (*p*)	−6.419 (0.001)	−6.707 (0.001)				
Rt. PPT (kg/cm^2^)	Pretest	2.30 ± 0.43	2.24 ± 0.31	0.510 (0.614)			
	Post-test	2.68 ± 0.44	2.34 ± 0.25				
	Mean difference	0.37 ± 0.28	0.10 ± 0.21		34.617 (0.001)	3.088 (0.088)	11.018 (0.002)
	95% CI for difference	(0.23 to 0.51)	(0.001 to 0.20)				
	*t* (*p*)	−5.685 (0.001)	−2.137 (0.047)				

Values are mean ± standard deviation; 95% CI = 95% confidence interval; Lt. = left; Rt. = right; PPT = pressure pain threshold.

**Table 3:** Comparison of postural assessment outcomes (*n* = 37).

Variable		Experimental Group (*n* = 18)	Control Group (*n* = 19)	*t* (*p*)	Time F (*p*)	Group F (*p*)	Time × Group F (*p*)
CVA (degrees, °)	Pretest	48.11 ± 3.32	46.68 ± 4.20	1.141 (0.262)			
	Post-test	52.11 ± 3.67	48.36 ± 3.98				
	Mean difference	4.00 ± 2.91	1.68 ± 1.60		54.984 (0.001)	4.663 (0.038)	9.126 (0.005)
	95% CI for difference	(−5.44 to −2.55)	(−2.45 to −0.91)				
	*t* (*p*)	−5.831 (0.001)	−4.587 (0.001)				
STD (degrees, °)	Pretest	4.22 ± 1.66	4.00 ± 1.73	0.397 (0.693)			
	Post-test	1.38 ± 0.50	3.00 ± 1.47				
	Mean difference	2.83 ± 1.72	1.00 ± 1.85		42.256 (0.001)	3.501 (0.070)	9.665 (0.004)
	95% CI for difference	(1.97 to 3.69)	(0.10 to 1.89)				
	*t* (*p*)	6.974 (0.001)	2.349 (0.030)				
Lt. RSP (mm)	Pretest	86.72 ± 11.29	85.15 ± 6.54	0.519 (0.607)			
	Post-test	74.66 ± 11.44	79.00 ± 6.48				
	Mean difference	12.05 ± 12.66	6.15 ± 4.53		34.625 (0.001)	0.284 (0.598)	3.630 (0.065)
	95% CI for difference	(5.75 to 18.35)	(3.97 to 8.34)				
	*t* (*p*)	4.037 (0.001)	5.916 (0.001)				
Rt. RSP (mm)	Pretest	90.66 ± 10.24	88.68 ± 7.28	0.681 (0.500)			
	Post-test	78.00 ± 9.13	81.52 ± 7.52				
	Mean difference	12.66 ± 10.12	7.15 ± 6.70		49.811 (0.001)	0.099 (0.755)	3.846 (0.058)
	95% CI for difference	(7.62 to 17.70)	(3.92 to 10.38)				
	*t* (*p*)	5.306 (0.001)	4.655 (0.001)				

Values are mean ± standard deviation; 95% CI = 95% confidence interval; CVA = craniovertebral angle; Lt. = left; Rt. = right; STD = shoulder tilt degree; RSP = round shoulder posture.

**Table 4:** Comparison of functional assessment outcomes (*n* = 37).

Variable		Experimental Group (*n* = 18)	Control Group (*n* = 19)	*t* (*p*)	Time F (*p*)	Group F (*p*)	Time × Group F (*p*)
NDI (scores)	Pretest	8.66 ± 5.72	8.47 ± 5.59	0.104 (0.262)			
	Post-test	2.50 ± 2.66	3.15 ± 2.63				
	Mean difference	6.16 ± 4.74	5.31 ± 3.30		73.719 (0.001)	0.032 (0.858)	0.405 (0.529)
	95% CI for difference	(3.80 to 8.52)	(3.72 to 6.90)				
	*t* (*p*)	5.516 (0.001)	7.020 (0.001)				
CKCUEST (times)	Pretest	18.00 ± 2.76	18.00 ± 3.38	0.000 (1.000)			
	Post-test	28.00 ± 3.94	27.00 ± 3.62				
	Mean difference	10.00 ± 3.86	9.00 ± 2.96		283.467 (0.001)	0.257 (0.616)	0.785 (0.382)
	95% CI for difference	(−11.92 to −8.07)	(−10.42 to −7.57)				
	*t* (*p*)	−10.976 (0.001)	−13.241 (0.001)				

Values are mean ± standard deviation; NDI = neck disability index; CKCUEST = closed kinetic chain upper extremity stability test.

## Data Availability

Data are contained within the article.
